# Development and Validation of a Nomogram for Preoperative Prediction of Early Recurrence after Upfront Surgery in Pancreatic Ductal Adenocarcinoma by Integrating Deep Learning and Radiological Variables

**DOI:** 10.3390/cancers15143543

**Published:** 2023-07-08

**Authors:** Fei Xiang, Xiang He, Xingyu Liu, Xinming Li, Xuchang Zhang, Yingfang Fan, Sheng Yan

**Affiliations:** 1 Department of Hepatobiliary Pancreatic Surgery, Second Affiliated Hospital, Zhejiang University School of Medicine, Hangzhou 310003, China; 2 Department of Hepatobiliary Surgery I, Zhujiang Hospital, Southern Medical University, Guangzhou 510280, China; 3 Department of Radiology, Zhujiang Hospital, Southern Medical University, Guangzhou 510280, China

**Keywords:** pancreatic ductal adenocarcinoma, early recurrence, deep learning, computed tomography, nomogram

## Abstract

**Simple Summary:**

Early recurrence is common after curative resection for pancreatic ductal adenocarcinoma (PDAC). Patients with a high-risk of early recurrence may benefit from a neoadjuvant-first approach instead of an upfront surgery. In our study, a deep-learning model for predicting early recurrence was developed and validated. The results showed that the deep learning model outputs were an independent risk factors associated with early recurrence. Additionally, higher values of deep learning model outputs were significantly associated with worse recurrence-free survival in various subgroups and demonstrated more advanced tumor behaviors. The comprehensive nomogram that integrated the deep learning model outputs and independent radiological factors further improved the predictive performance. Our findings show that the deep learning-based nomogram could noninvasively predict early recurrence in PDAC patients, which may support clinical decision-making about upfront resection or neoadjuvant treatment strategies.

**Abstract:**

Around 80% of pancreatic ductal adenocarcinoma (PDAC) patients experience recurrence after curative resection. We aimed to develop a deep-learning model based on preoperative CT images to predict early recurrence (recurrence within 12 months) in PDAC patients. The retrospective study included 435 patients with PDAC from two independent centers. A modified 3D-ResNet18 network was used for a deep learning model construction. A nomogram was constructed by incorporating deep learning model outputs and independent preoperative radiological predictors. The deep learning model provided the area under the receiver operating curve (AUC) values of 0.836, 0.736, and 0.720 in the development, internal, and external validation datasets for early recurrence prediction, respectively. Multivariate logistic analysis revealed that higher deep learning model outputs (odds ratio [OR]: 1.675; 95% CI: 1.467, 1.950; *p* < 0.001), cN1/2 stage (OR: 1.964; 95% CI: 1.036, 3.774; *p* = 0.040), and arterial involvement (OR: 2.207; 95% CI: 1.043, 4.873; *p* = 0.043) were independent risk factors associated with early recurrence and were used to build an integrated nomogram. The nomogram yielded AUC values of 0.855, 0.752, and 0.741 in the development, internal, and external validation datasets. In conclusion, the proposed nomogram may help predict early recurrence in PDAC patients.

## 1. Introduction

Pancreatic ductal adenocarcinoma (PDAC) represents one of the most lethal malignancies with a five year survival rate of less than 10% [1,2]. Radical resection with adjuvant chemo(radio)therapy is considered the major therapy for treating PDAC. Even in patients with resectable PDAC, recurrence occurs in approximately 80%, with 50% occurring within one year [3,4]. Neoadjuvant chemo(radio)therapy has been reported may decrease recurrence and improve survival, especially for borderline resectable (BRPC) and locally advanced PDAC (LAPC) [5,6,7]. Therefore, identifying patients with a high recurrence risk is essential, as these patients may benefit from a neoadjuvant-first approach instead of an upfront surgery.

Currently, predicting the recurrence of PDAC is mainly based on multiple clinicopathological factors. Postoperative pathological factors, such as lymph node metastases and tumor differentiation, are the most reported independent predictors of recurrence [8,9,10]. However, pathological indicators that may not allow preoperative clinical decision-making can only be acquired after surgery. Several preoperative score indicators or clinical factors, such as Glasgow prognostic score, carbohydrate antigen 19-9 (CA19-9), and platelet-to-lymphocyte ratio, have been reported to be associated with postoperative recurrence [11,12,13]. Nevertheless, these factors have not yet been the subject of widespread recognition or validation.

Imaging methods, including computed tomography (CT) and magnetic resonance imaging (MRI), are widely used for PDAC diagnosis, staging, and resectability evaluation. Furthermore, some studies [14,15,16] reported that some imaging characteristics, such as suspicious metastatic lymph nodes, hypodense tumor in the portal venous phase, peripancreatic tumor infiltration, tumor necrosis, and presence of pancreatitis or pseudocyst, were associated with postoperative tumor recurrence. Recently, there has been growing interest in applying deep learning for prognosis prediction from cancer imaging. Deep learning is a powerful approach to the extraction of information from medical images and has shown promise for survival prediction in PDAC patients [17,18,19]. However, to the best of our knowledge, deep learning methods have not been well-evaluated for predicting recurrence in PDAC patients.

Therefore, we aimed to develop a deep learning model based on preoperative contrast-enhanced CT (CECT) images for the prediction of early recurrence (ER) after upfront surgery in patients with PDAC. Moreover, a comprehensive preoperative nomogram was established by integrating the deep learning model outputs and radiological variables.

## 2. Materials and Methods

### 2.1. Patients 

Our study recruited patients from Zhejiang University School of Medicine Affiliated Second Hospital (Center 1, for developing models and internal validation) and Southern Medical University Affiliated Zhujiang Hospital (center 2, for independent external validation). The criteria for inclusion were as follows: histologically confirmed PDAC and contrast-enhanced CT performed within 1 month before surgery. Exclusion criteria included the following: use of neoadjuvant therapy, including radiotherapy, chemotherapy, or other treatments, unavailability of preoperative computed tomography (CT) or suboptimal image quality, 90-day postoperative mortality, coexisting other malignancy, no visible mass at CT, and multiple synchronous PDAC. Patients were also excluded if their records were incomplete or had less than 12 months of follow-up without recurrence or death. The detailed process is shown in Figure S1.

### 2.2. Outcomes and Data Collection

Baseline characteristics, including age, sex, liver function test, and serum CA19-9 level, were collected. Preoperative CT imaging was used to assess vascular involvement and tumor size. Venous involvement comprised the portal vein, superior mesenteric vein, and spleen vein. The arterial involvement included the coeliac trunk, superior mesenteric artery, common hepatic artery, and spleen artery. The T and N stages were determined based on preoperative CT images according to the 8th AJCC TNM staging system. R0 resection was defined as the absence of identifiable tumor cells within 1 mm of the resection margin. All patients were followed every month for the first six months after surgery for adjuvant chemo(radio)therapy, every three months for the following 1.5 years, and once a year after that. At each follow-up, serum CA19-9 levels were measured, and imaging (contrast-enhanced CT or MRI) was performed. ER was defined as recurrence within 12 months after surgery.

### 2.3. CT Acquisition and Image Processing

**Figure 1:**
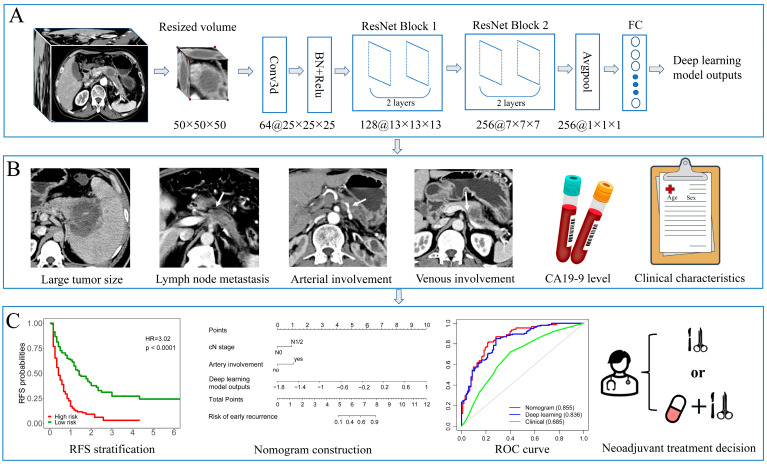
The workflow of this study. (**A**) The primary tumor was cropped and resized to a uniform size (50 × 50 × 50) as the input to a 3D deep learning network. The deep learning model was constructed using a modified 3D-ResNet18 framework, and the outputs were used for integrated nomogram construction. (**B**) Preoperative factors such as tumor size, lymph node metastasis, venous invasion, artery invasion, CA19-9 level, and baseline clinical characteristics were inputted into univariable and multivariate logistics regression to select independent factors for nomogram and clinical modeling. (**C**) High-risk group patients showed significantly worse recurrence-free survival in the Kaplan–Meier analysis. A nomogram was created by incorporating independent radiological factors and deep learning model outputs. The ROC curve was used to compare the predictive performance of developed models. The nomogram can support shared decision-making regarding upfront resection or neoadjuvant treatment strategies.

Figure 1 shows the workflow of this study. All patients received a contrast-enhanced CT scan prior to surgery. This study used images of the portal venous phase for deep learning model construction. CT acquisition protocols of the two centers can be found in the Supplementary Materials.

The image intensity values were truncated from −125 to 225 HU (window width: 250 HU, window level: 50 HU) and then resampled to a resolution of 1 × 1 × 3 mm^3^ using spline interpolation to decrease the variability between scans. Finally, each pixel value was standardized to the range of [0, 1]. The 3D primary tumor was manually segmented using the ITK-SNAP software (version 3.6.0) on the portal venous-phase CT images by two radiologists (X.M.L, X.C.Z, with 10 and 20 years of work experience) in consensus. The primary tumor was then cropped and resized to a uniform size (50 × 50 × 50) as the input to a 3D deep-learning network.

### 2.4. Deep Learning Model Development

We used a modified 3D-ResNet-18 to develop the CT-based deep learning model. The channel of the first convolutional layer of the network was modified from a three-channel into a single-channel, ensuring that the network can accept gray images as input. In addition, the 3D convolutional kernel with size (3 × 3 × 3) instead of (7 × 7 × 7) was used for a relatively small size of the input. Then two ResNet layers with 2 and 3 basic blocks were appended to increase network depth. Finally, the output layer was modified to classify patients into two classes (with or without ER). The outputted conditional probabilities indicated the individual recurrence risk used for integrated nomogram construction (The code can be found at https://github.com/fatfeifei/PDAC_recurrence_prediction (accessed on 15 May 2023)).

During the model’s training, all inputted 3D images were augmented using the torchIO (version 0.18.86), such as translation, rotation, or shearing, and the magnitude of the operations. Patients in Center 1 were randomly split into development and internal validation datasets (7:3 ratio). To minimize the loss, the Adam optimizer was used with a learning rate of 1 × 10^−4^. The loss function was binary cross-entropy. The training was aborted when the loss in the validation dataset did not decrease for 10 epochs.

### 2.5. Performance Evaluation in Different Subgroups

Patients were classified into high-risk and low-risk groups with the median value of the output probabilities of the deep learning model in the development dataset as the cutoff. Clinicopathological characteristics and surgery details were compared between high- and low-risk groups. 

Furthermore, we classified all patients into clinicopathological subgroups based on tumor location, age (<70 vs. ≥70 y), sex (male vs. female), TBIL level (≤21 vs. >21 U/mL), CA19-9 level (<120 vs. ≥120 U/mL), pT stage (T1/2 vs. T3/4), pN stage (N0 vs. N1/2), and tumor differentiation (well vs. moderate/poor). The performance of deep learning model outputs in different subgroups was assessed using Kaplan–Meier method.

### 2.6. Nomogram and Clinical Model Construction

The selection of significant risk factors for ER was performed using logistic regression analysis. First, the deep learning model outputs and preoperative clinical factors were analyzed using univariable logistic regression analysis. The multivariate logistics regression analysis included the factors with a *p*-value of less than 0.1. Next, risk factors were selected using stepwise backward elimination based on the Akaike information criterion (AIC). Better model fit was indicated by a lower Akaike information criterion. The selected variables were then used to generate the nomogram. Then multivariate logistic regression was applied repeatedly without deep learning model outputs to develop the clinical model. Model discrimination was assessed and compared via area under the receiver operating curve (AUC). Calibration curves and Hosmer–Lemeshow tests were used to assess the agreement between the nomogram prediction and the actual observed rate. 

### 2.7. Statistical Analysis

Data analyses were performed using Python (version 3.8.0) and R software (version 4.0.3). The deep learning model was implemented based on PyTorch (version 1.10.2). Categorical variables were compared using Chi-square or Fisher’s exact test. The “rms” package (https://github.com/harrelfe/rms (version 6.7-0 accessed on 8 May 2023)) was used for logistic regression analysis and nomogram and calibration curve plotting. The AUC values were compared using DeLong’s test with “pROC” package [20]. The Hosmer–Lemeshow test was implemented using “generalhoslem” package (https://github.com/matthewjay15/generaLhosLem (accessed on 3 June 2019)).

## 3. Results

### 3.1. Patient Characteristics

A total of 368 PDAC patients in Center 1 were selected and randomized to the development (*n* = 257) and internal validation (*n* = 111) datasets. The external validation dataset consisted of 67 patients from Center 2. Table 1 summarizes the baseline clinical characteristics, showing no significant differences between the datasets except sex, diabetes, organ involvement, and perineural invasion. The median follow-up was 9.0 months (interquartile range [IQR]: 4.0–16.0) in Center 1 and 10.0 months (IQR: 3.0–20.0) in Center 2. At the last follow-up, 275/368 (74.7%) and 44/67 (65.7%) patients in each center had experienced recurrence.

### 3.2. Development and Validation of Deep Learning Model

**Figure 2:**
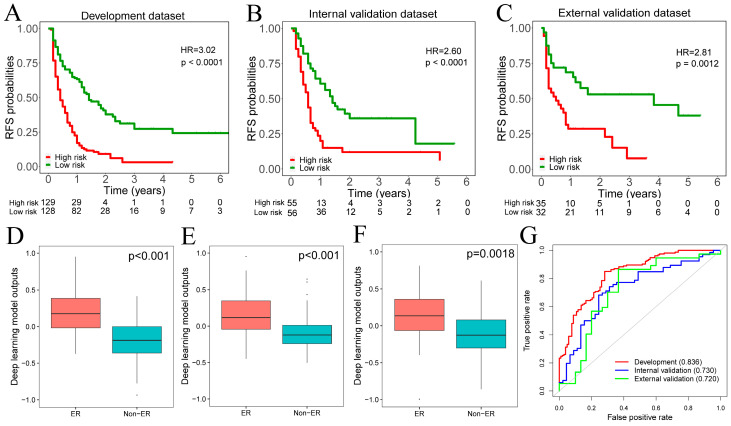
(**A**–**C**). The Kaplan–Meier curves showed that the high-risk group had a significantly worse RFS than the low-risk group across development, internal, and external validation datasets. (**D**–**F**). Patients who experienced ER had significantly higher deep learning model outputs across development, internal, and external validation datasets. (**G**). The ROC analysis demonstrated that the deep learning model outputs achieved satisfactory prediction performances across development, internal, and external validation datasets. RFS, recurrence-free survival; ER, early recurrence.

The range of deep learning model outputs was (−1.68–1.01) with a median value of 0.18 in the development dataset. According to the median value of deep learning model outputs, all patients were assigned to high-risk (≥median value) and low-risk (<median value) groups. Kaplan–Meier analyses depicted that patients in the high-risk group had lower recurrence-free survival (RFS) than in the low-risk group among development, internal, and external validation datasets (Figure 2A–C). Meanwhile, across the development, internal, and external validation datasets, patients who experienced ER had higher values of deep learning model outputs than those without (Figure 2D–F). The ROC curve analysis demonstrated that the deep learning model provided AUC values of 0.836, 0.736, and 0.720 in the development, internal, and external validation datasets for predicting ER, respectively (Figure 2G).

Clinicopathological characteristics were also compared between the high-risk and low-risk groups.

The high-risk group showed more aggressive tumor behavior, including higher CA19-9 level, advanced T stage, lymph node metastasis, larger tumor size, and poorer tumor differentiation (all *p* < 0.005). Additionally, in patients with vascular involvement, adjacent organ invasions occurred more frequently in high-risk groups (all *p* < 0.005). The detailed comparison results are provided in Table S1.

**Figure 3:**
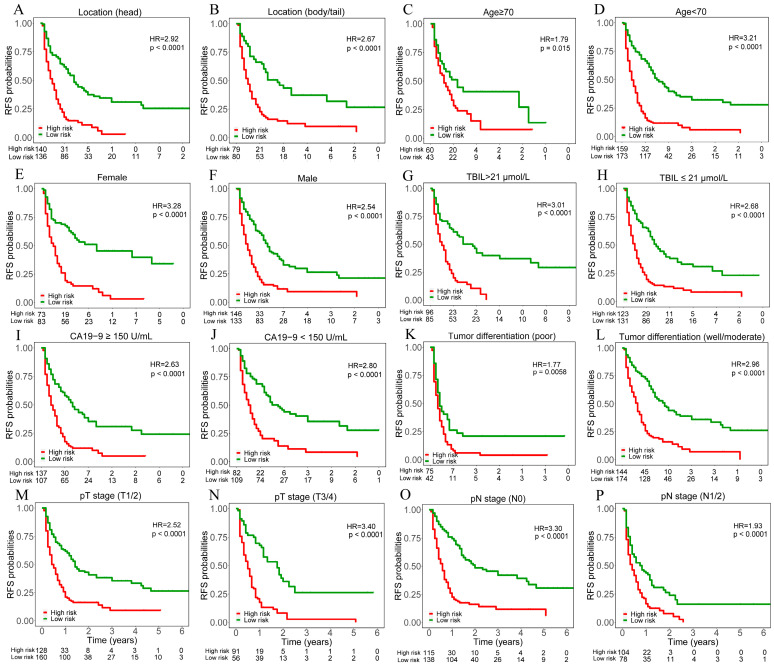
Kaplan–Meier curves of recurrence free survival (RFS) for patients in different risk subgroups stratified by the median value of deep learning model outputs. Patients were divided into different risk subgroups according to tumor location ((**A**). head, (**B**). body/tail), age ((**C**). ≥70, (**D**). <70), sex ((**E**). female, (**F**). male), TBIL level ((**G**). >21 μmol/L, (**H**). ≤21 μmol/L), CA19-9 level ((**I**). ≥150 U/mL, (**J**). <150 U/mL), tumor differentiation ((**K**). poor, (**L**). well/moderate), pT stage ((**M**). T1/2, (**N**). T3/4), pN stage ((**O**). N0, (**P**). N1/2). The *X*-axis of all curves is the year and ranges from 0 to 6.

Moreover, we tested the prognostic value of the binary deep learning model outputs within each subgroup of patients. The results demonstrated significant differences in RFS between high versus low-risk patients in all subgroups (Figure 3).

### 3.3. Nomogram and Clinical Modeling

**Figure 4:**
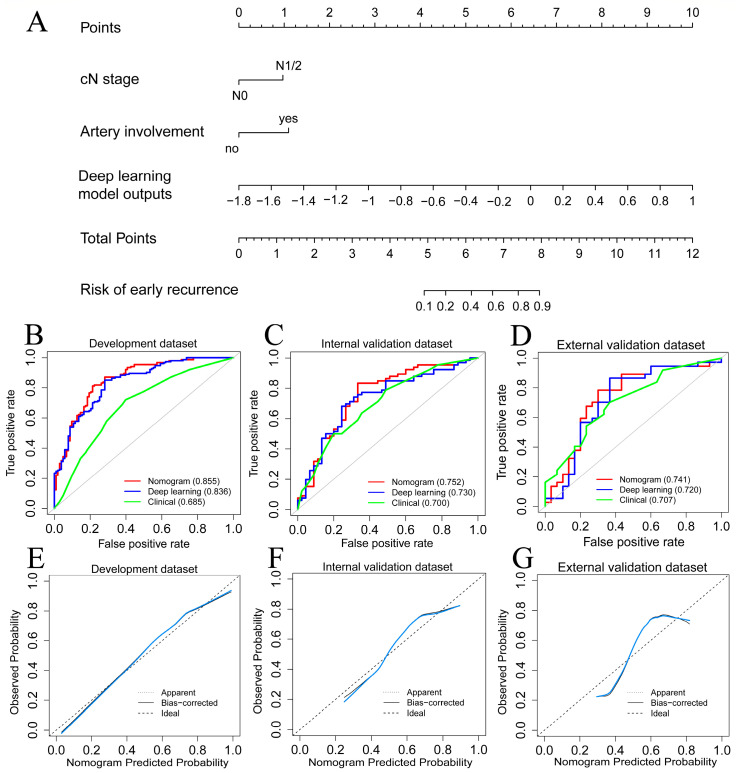
(**A**). The nomogram was constructed by incorporating deep learning model outputs and two independent radiological factors. (**B**–**D**). The ROC curve of developed models in the development, internal, and external validation datasets. (**E**–**G**). Calibration curves (bule lines) of the nomogram in the development, internal, and external validation datasets.

The multivariable logistic analysis of ER with deep learning model outputs and preoperative clinical factors is shown in Table 2. The results revealed three independent preoperative factors for ER: arterial involvement (OR: 2.207; 95% CI: 1.043, 4.873; *p* = 0.043), cN1/2 stage (OR: 1.964; 95% CI: 1.036, 3.774; *p* = 0.040), and deep learning model outputs (odds ratio [OR]: 1.675; 95% CI: 1.467, 1.950; *p* < 0.001). The nomogram was constructed to predict ER by incorporating these independent risk factors (Figure 4A). On the other hand, by eliminating deep learning model outputs, four clinical variables with the smallest AIC were selected to develop the clinical model (Tables S2 and S3): CA19-9 level ≥ 150 U/mL (OR: 1.766; 95% CI: 1.047, 2.966; *p* = 0.034), CT reported tumor size ≥ 3.0 cm (OR: 1.734; 95% CI: 1.108, 2.974; *p* = 0.044), cN1/2 stage (OR: 1.982; 95% CI: 1.164, 3.409; *p* = 0.012), and arterial involvement (HR: 2.145; 95% CI: 1.145, 4.157; *p* = 0.020).

### 3.4. Model Performance Comparison

Table 3 summarized the predictive performance of all developed models. The nomogram yielded AUC values of 0.855, 0.752, and 0.741 in the development, internal, and external validation datasets, respectively (Figure 4B–D). DeLong’s test showed that the nomogram model outperformed the clinical model in the development dataset (*p* < 0.001) but was comparable in the internal (*p* = 0.293) and external (*p* = 0.364) datasets. The calibration curves of the nomogram showed good agreements in the development, internal, and external validation datasets, respectively (Figure 4E–G). The Hosmer–Lemeshow test further indicated the good calibration of the nomogram for all datasets (*p* = 0.915, 0.797, and 0.367 for the development, internal, and external validation datasets). 

## 4. Discussion

This study developed a CT-based deep-learning model for predicting ER after upfront surgery in patients with PDAC. A subgroup analysis demonstrated that the median value of deep learning model outputs could stratify PDAC patients into high and low-risk groups with significantly different prognoses. In addition, the combined nomogram integrating the deep learning model outputs and radiological variables further enhanced predictive abilities.

Biomedical images contain information that reflects underlying tumor pathophysiology. Machine learning model construction based on image features has been increasingly explored for identifying high-risk PDAC patients. Radiological characteristics, such as suspicious lymph node metastasis and peripancreatic tumor infiltration, are well-known high-risk imaging features associated with early recurrence in PDAC patients [14,21]. Quantitative image analyses, such as radiomics and deep learning, represent a novelty approach contributing to decision support in oncology [22,23]. Radiomics features, such as kurtosis and grey-level non-uniformity, have the potential to indicate the presence of tumor heterogeneity. Studies [16,24] indicated that high values of these two features were linked to ER in PDAC. In addition, Sandrasegaran et al. [25] reported that high kurtosis, and the mean value of positive pixels were predictors of worse overall survival in PDAC patients. Unlike radiomics, which relied on domain expertise to define features and needed to undergo feature extraction processes, feature selection, and machine learning modeling, deep learning is the end-to-end one-step process that automatically learns effective features and simultaneously outputs predicted probability values. Lee et al. [19] developed an ensemble model that integrated a series of deep learning and machine learning models that showed better performance in predicting one-year RFS in PDAC patients than the AJCC staging system. Yao et al. [18] proposed a 3D Convolutional LSTM network using multiple CECT phase data for predicting overall survival in PDAC patients. The multivariable analysis revealed that the deep learning score strongly predicted PDAC survival. 

In our study, the modified 3D-ResNet-18 was used as the backbone network for deep learning model construction. The ResNet architecture enables the depth of the network to increase without degradation and can therefore improve the representation of its learning ability [26]. The multivariable logistic regression and the nomogram plot showed that the deep learning model outputs were the strongest predictor among all preoperative clinical variables. In addition, we further dichotomized all patients into high- and low-risk groups based on the median deep learning model outputs. The discrimination ability of the binary deep learning model outputs was validated in various clinical subgroups. In addition, when clinicopathological variables were compared between high- and low-risk groups, the high-risk group patients demonstrated more aggressive tumor behavior. These results suggested that the deep learning model outputs may have the potential as a useful preoperative prognostic biomarker. 

The multivariable logistic regression also found that artery involvement and cN stage were independent factors that synergistically predicted the ER. PDAC with artery involvement has been reported in association with advanced tumor characteristics, lower R0 resection rate, and higher recurrence rate [27,28,29]. With respect to the cN stage, a number of studies [14,21,30] reported that the detection of lymph node metastasis preoperative CT scans (cN1/2) was a useful predicter of ER. The nomogram integrating the deep learning model outputs and these two radiological variables showed an improved predictive performance over the clinical model. Notably, nomogram construction variables were all derived from preoperative CT imaging. The results indicated that combining deep learning and conventional radiological variables can integrate both advantages.

Our study has some limitations. First, the deep learning model was trained and validated on a relatively small dataset. Second, the retrospective design may have selection bias and unknown confounding factors. Third, the primary tumor was manually segmented. Although manual segmentation is more accurate than automatic, the process is laborious and time-consuming. Therefore, an automatic segmentation and prediction deep learning model trained in a large dataset may improve efficiency and prediction performance.

## 5. Conclusions

We proposed a preoperative deep learning model and an integrated nomogram based on preoperative CT images that can noninvasively predict ER in PDAC patients. This may aid clinical decision-making regarding upfront resection or neoadjuvant treatment strategies in PDAC patients.

## Figures and Tables

**Table 1:** Characteristics of patients in the development, internal validation, and external validation datasets.

Characteristic	Development Dataset (*n* = 257)	Internal Validation Dataset (*n* = 111)	External Validation Dataset (*n* = 67)	*p* Value
Age, years				0.946
≥70	62 (24)	25 (22)	16 (24)	
<70	195 (76)	86 (78)	51 (76)	
Sex				0.044
Female	87 (34)	36 (32)	33 (49)	
Male	170 (66)	75 (68)	34 (51)	
Diabetes				0.004
No	197 (77)	84 (76)	38 (57)	
Yes	60 (23)	27 (24)	29 (43)	
Alb				0.173
≥35 U/mL	221 (86)	99 (89)	53 (79)	
<35 U/mL	36 (14)	12 (11)	14 (21)	
TBIL				0.088
>21 μmol/L	109 (42)	38 (34)	34 (51)	
≤21 μmol/L	148 (58)	73 (66)	33 (49)	
CA19-9				0.222
≥150 U/mL	139 (54)	70 (63)	35 (44)	
<150 U/mL	118 (46)	41 (37)	32 (56)	
CT tumor size				0.134
≥3.0 cm	142 (55)	50 (45)	31 (46)	
<3.0 cm	115 (45)	61 (55)	36 (54)	
Location				0.088
Head	164 (64)	63 (57)	49 (73)	
Body/Tail	93 (36)	48 (43)	18 (27)	
cT stage (AJCC 8th edition)				0.184
cT1-T2	195 (76)	74 (67)	48 (72)	
cT3-T4	62 (24)	37 (33)	19 (28)	
cN stage (AJCC 8th edition)				0.677
cN0	143 (56)	61 (55)	41 (61)	
cN1-N2	114 (44)	50 (45)	26 (39)	
Vascular involvement on CT imaging				0.740
No	143 (56)	57 (52)	37 (55)	
Arterial	17 (6)	7 (6)	3 (5)	
Venous	46 (18)	16 (14)	12 (18)	
Both	51 (20)	31 (28)	15 (22)	
Organ involvement on CT imaging				<0.001
No	223 (87)	93 (84)	35 (52)	
Yes	34 (13)	18 (16)	32 (48)	
Resection Margin				0.553
R0	238 (93)	99 (89)	61 (91)	
R1	19 (7)	12 (11)	6 (9)	
pT stage (AJCC 8th edition)				0.154
pT1-T2	179 (70)	66 (60)	43 (64)	
pT3-T4	78 (30)	45 (40)	24 (36)	
pN stage (AJCC 8th edition)				0.077
pN0	139 (54)	74 (67)	40 (60)	
pN1-N2	118 (46)	37 (33)	27 (40)	
Perineural invasion				0.021
No	49 (19)	12 (11)	5 (7)	
Yes	208 (81)	99 (89)	62 (93)	
Tumor differentiation				0.666
Well	31 (12)	10 (9)	6 (9)	
Moderate	161 (63)	66 (60)	44 (66)	
Poor	65 (25)	35 (31)	17 (25)	

Abbreviations: Alb albumin, TBIL total bilirubin, CA-199 carbohydrate antigen 19-9, AJCC, American Joint Committee on Cancer. Note: Data are numbers of patients, with percentages in parentheses. Chi-squared or Fisher’s exact tests, were used to compare the differences in categorical variables.

**Table 2:** Univariate and multivariate logistic analyses of risk factors for early recurrence.

Variables	Univariate Analysis			Multivariate Analysis		
	OR	95% CI	*p*-Value	OR	95% CI	*p*-Value
Age (≥70 vs. <70)	1.417	0.779–2.576	0.253			
Sex (Male vs. Female)	1.166	0.690–1.972	0.566			
Diabetes (yes vs. no)	1.004	0.557–1.811	0.989			
Alb (<35 vs. ≥35)	1.400	0.666–2.943	0.375			
TBIL (>21 vs. ≤21)	1.196	0.720–1.985	0.489			
CA19-9 (≥150 vs. <150)	1.768	1.068–2.927	0.027			
CT tumor size (≥3.0 vs. <3 cm)	1.960	1.173–3.277	0.010			
Location (head vs. body/tail)	1.297	0.774–2.175	0.324			
cT stage (cT3/4 vs. cT1/2)	2.316	1.227–4.371	0.010			
cN stage (cN1/2 vs. cN0)	2.194	1.307–3.684	0.003	1.964	1.036–3.774	0.040
Arterial involvement (yes vs. no)	2.505	1.349–4.652	0.004	2.207	1.043–4.870	0.043
Venous involvement (yes vs. no)	1.742	1.027–2.955	0.040			
Organ involvement (yes vs. no)	2.024	0.903–4.536	0.087			
Deep learning model outputs (per 0.1 increase)	1.699	1.477–1.954	<0.001	1.675	1.467–1.950	<0.001

Abbreviations: OR, odds ratio; CI, confidence interval.

**Table 3:** Performance of developed models in predicting early recurrence.

Models	Dataset	AIC	AUC (95% CI)	Accuracy (95% CI)	Sensitivity (95% CI)	Specificity (95% CI)
Nomogram	Development	243.674	0.855 (0.787–0.886)	79.0 (73.5–83.8)	87.0 (80.7–91.9)	67.0 (57.0–76.0)
	Internal validation	133.613	0.752 (0.657–0.848)	73.9 (64.7–81.8)	83.3 (72.1–91.4)	60.0 (44.3–74.3)
	External validation	86.133	0.741 (0.615–0.867)	73.1 (60.9–83.2)	78.4 (61.8–90.2)	66.7 (47.2–82.7)
Deep learning	Development	249.189	0.836 (0.787–0.886)	78.2 (72.7–83.1)	82.5 (75.5–88.1)	71.8 (62.1–80.3)
	Internal validation	137.467	0.730 (0.633–0.826)	72.1 (62.8–80.2)	75.8 (63.6–85.5)	66.7 (51.0–80.0)
	External validation	89.323	0.720 (0.589–0.851)	70.1 (57.7–80.7)	73.0 (55.9–86.2)	66.7 (47.2–82.7)
Clinical	Development	330.916	0.685 (0.618–0.752)	66.5 (60.4–72.3)	77.3 (69.8–83.6)	50.5 (40.5–60.5)
	Internal validation	139.637	0.700 (0.601–0.799)	67.6 (58.0–76.1)	78.8 (67.0–87.9)	51.1 (35.8–66.3)
	External validation	86.603	0.707 (0.583–0.831)	67.2 (54.6–78.2)	70.3 (53.0–84.1)	63.3 (43.9–80.1)

Abbreviations: AIC akaike information criterion, AUC area under the receiver operating curve, CI confidence interval.

## Data Availability

CT images and clinical data are available upon reasonable request from the corresponding authors. The codes for image processing and deep learning model construction have been uploaded to https://github.com/fatfeifei/PDAC_recurrence_prediction (accessed on 15 May 2023).

## Supplementary Materials

The following supporting information can be downloaded at: https://www.mdpi.com/article/10.3390/cancers15143543/s1, Figure S1: Inclusion criteria and exclusion criteria; Supplementary Method S1. CT image acquisition protocol of two centers; Table S1. The comparison results between high-risk and low-risk groups; Table S2. Univariate and multivariate logistic analyses of risk factors for recurrence without deep learning model outputs; Table S3. Selection process for the clinical model.
