# Supplementary material for: Development and Validation of a Nomogram for Preoperative Prediction of Early Recurrence after Upfront Surgery in Pancreatic Ductal Adenocarcinoma by Integrating Deep Learning and Radiological Variables
Source: Cancers (Basel). 2023 Jul 8;15(14):3543. doi: 10.3390/cancers15143543 (PMC10377149; doi:10.3390/cancers15143543)
Supplement: Supplementary file 1 [file cancers-15-03543-s001.zip › cancers-2427256-supplementary.pdf]

Supplementary materials

1.Figure S1. Inclusion criteria and exclusion criteria
2.Supplementary Method S1. CT image acquisition protocol of two centers
3.Table S1. The comparison results between high-risk and low-risk groups
4.Table S2. Univariate and multivariate logistic analyses of risk factors for recurrence without deep learning model outputs
5. Table S3. Selection process for the clinical model.

1.Figure S1. Inclusion criteria and exclusion criteria

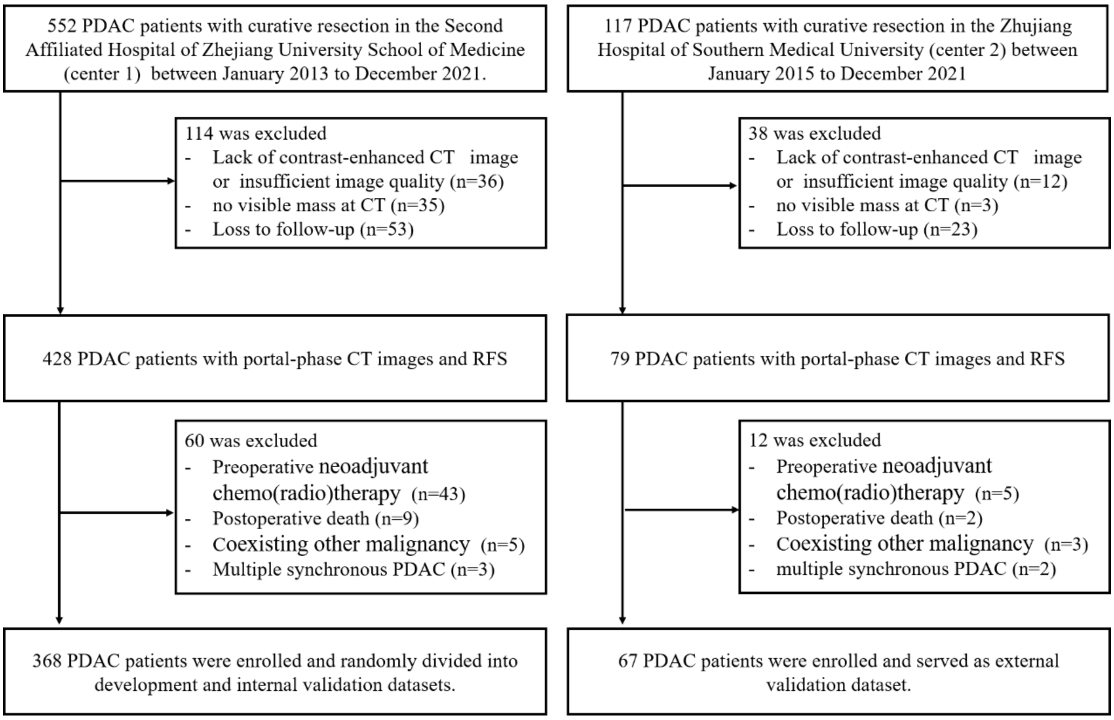

2. Supplementary Method S1. CT image acquisition protocol of two centers

CT examinations performed in the Second Affiliated Hospital of Zhejiang University School of Medicine using SOMATOM Perspective (Siemens Healthineers), SOMATOM Definition Flash (Siemens Healthineers), and SOMATOM Definition AS (Siemens Healthineers). The following scanning parameters were used: tube current, 10 to 644 mA; tube voltage, 100 to 130 KV; slice thickness, 5 mm; detector collimation: 8×2.5 mm or 64×0.625mm; field of view, 350×350 mm; matrix, 388×388. For dynamic contrast-enhanced CT imaging, about 1.3 mL/kg of body weight bolus of iodinated contrast material (Iohexol, GE Healthcare, USA) was injected intravenously at a rate of 3.0 mL/s. The arterial phase (AP) and portal venous phase (PP) were acquired at 40 s, 72 s after the start of contrast injection, respectively.

All patients underwent CT examinations performed in the Zhujiang Hospital of Southern Medical University using a Philips Brilliance 64-MDCT scanner. The scanning parameters included a voltage of 120 kV, a current of 200 mA, a rotation time of 0.5 seconds, a detector combination of 0.625×64 mm, a pitch of 0.894 and a bed speed of 47.5 mm/s. After non-enhanced CT, dynamic abdominal triphasic

tomography and thin-slice scanning were performed for patients after an intravenous contrast agent, namely, non-ionic iopamiro, was administered. Each patient received 80-100 mL of vascular contrast agents at a rate of 5 mL/s with a pump injector. Arterial-phase scanning was achieved by contrast agent tracing. Specifically, the scanning was automatically triggered 8 seconds after the vascular CT value in the diaphragmatic section of the abdominal aorta reached 100 HU. Portal venous phase scanning was initiated per the same criterion but with a 60-second delay.

3. Table S1. The comparison results between high-risk and low-risk groups

**Characteristic**	**High risk group (n=219)**	**Low risk group (n=216)**	**P value**
Age, years			0.066
≥70	60 (27)	43 (20)	
< 70	159 (73)	173 (80)	
Sex			0.268
Female	73 (33)	83 (38)	
Male	146 (67)	133 (62)	
Diabetes			0.573
No	158 (72)	161 (75)	
Yes	61 (28)	55 (25)	
Alb			0.624
≥ 35 U/mL	186 (85)	187 (87)	
<35 U/mL	33 (15)	29 (13)	
TBIL			0.343
>21 U/mL	96 (44)	85 (39)	
<=21 U/mL	123 (56)	131 (61)	
CA19-9			**0.006**
≥ 150 U/mL	137 (63)	107 (49)	
<150 U/mL	82 (37)	109 (51)	
CT tumor size			**0.002**
≥ 3.0 cm	133 (61)	99 (46)	
< 3.0 cm	86 (39)	117 (54)	
Location			0.835
Head	140 (64)	136 (63)	
Body/Tail	79 (36)	80 (37)	
cT stage (AJCC 8th edition)			**< 0.001**
cT1-T2	139 (64)	178 (82)	
cT3-T4	80 (36)	38 (18)	
cN stage (AJCC 8th edition)			**0.001**
cN0	106 (48)	139 (64)	
cN1-N2	113 (52)	77 (36)	
Arterial involvement			**0.002**
No	142 (65)	169 (78)	
Yes	77 (35)	47 (22)	

Venous involvement			**0.002**
No	117 (53)	147 (68)	
Yes	102 (47)	69 (32)	
Organ involvement on CT imaging			**< 0.001**
No	157 (72)	194 (90)	
Yes	62 (28)	22 (10)	
Resection Margin			**0.065**
R0	195 (89)	203 (94)	
R1	24 (11)	13 (6)	
pT stage (AJCC 8th edition)			**0.001**
pT1-T2	128 (58)	160 (74)	
pT3-T4	91 (42)	56 (26)	
pN stage (AJCC 8th edition)			**0.016**
pN0	115 (53)	138 (64)	
pN1-N2	104 (47)	78 (36)	
Perineural Invasion			0.096
No	27 (12)	39 (18)	
Yes	192 (88)	177 (82)	
Tumor differentiation			**0.003**
Well	10 (5)	37 (17)	
Moderate	134 (61)	137 (63)	
Poor	75 (75)	42 (20)	

4. Table S2. Selection process for the clinical model.

Feature set	AIC
CA19-9 + cN stage + cT stage + CT reported size + Organ involvement + Venous involvement + Artery involvement	336.02
CA19-9 + cN stage + cT stage + CT reported size + Organ involvement + Artery involvement	334.05
CA19-9 + cN stage + CT reported size + Organ involvement + Artery involvement	332.48
CA19-9 + cN stage + CT reported size + Artery involvement	330.92

5. Table S3. Univariate and multivariate logistic analyses of risk factors for early recurrence without deep learning model outputs.

Variables	Univariate analysis			Multivariate analysis		
	OR	95% CI	P-value	OR	95% CI	P-value
Age (≥ 70 vs < 70)	1.417	0.779-2.576	0.253			
Sex (Male vs Female)	1.166	0.690-1.972	0.566			
Diabetes (yes vs no)	1.004	0.557-1.811	0.989			
Alb (35 < vs ≥ 35)	1.400	0.666-2.943	0.375			
TBIL (≥ 21 vs < 21)	1.196	0.720-1.985	0.489			
CA-199 (≥ 150 vs < 150)	1.768	1.068-2.927	0.027	1.766	1.047-2.966	0.034
CT tumor size (≥ 3.0 vs < 3 cm)	1.960	1.173-3.277	0.010	1.734	1.108-2.974	0.044
Location (head vs body/tail)	1.297	0.774-2.175	0.324			
cT stage (cT3/4 vs cT1/2)	2.316	1.227-4.371	0.010			
cN stage (cN1/2 vs cN0)	2.194	1.307-3.684	0.003	1.982	1.164-3.409	0.012
Arterial involvement (yes vs no)	2.505	1.349-4.652	0.004	2.145	1.145-4.157	0.020
Venous involvement (yes vs no)	1.742	1.027-2.955	0.040			
Organ involvement (yes vs no)	2.024	0.903-4.536	0.087			

Abbreviations: OR, odds ratio; CI, confidence interval.
